# Minimally Invasive Liver Surgery: A Snapshot from a Major Dutch HPB and Transplant Center

**DOI:** 10.1007/s00268-022-06754-z

**Published:** 2022-09-26

**Authors:** Rebecca Marino, Pim B. Olthof, Hong J. Shi, Khe T. C. Tran, Jan N. M. Ijzermans, Türkan Terkivatan

**Affiliations:** 1 Hepatobiliary Surgery Division, IRCCS San Raffaele Hospital, Via Olgettina 60, 20132 Milan, Italy; 2 Department of Surgery, Division of HPB & Transplant Surgery, University Medical Centre Rotterdam, Dr. Molewaterplein 40, Erasmus MC, 3015 GD Rotterdam, The Netherlands

## Abstract

**Background:**

Minimally invasive liver surgery (MILS) has been progressively adopted on a nationwide scale. The aim of this study is to investigate MILS implementation in a high-volume Dutch hepato-pancreato-biliary and transplant center, which is considered a moderate to low-volume center from a European standpoint.

**Methods:**

All patients who underwent MILS at Erasmus Medical Center between April 2010 and December 2021 were retrospectively reviewed. Patients’ surgical outcomes were compared after stratification according to resections’ difficulty and liver cirrhosis.

**Results:**

A total of 212 cases were included. Major liver resections were performed in 24 patients (11%), while minor resections were performed in 188 patients (89%). Among those, 177 (94%) resections were classified as technically minor and 11 (6%) as technically major. Major morbidity was reported in 14/177 patients (8%) after technically minor resections and in 3/24 patients (13%) after major resections. Anatomically and technically major resections had higher intraoperative blood losses (425 (0–2100) vs. 240 (50–110) vs. 100 (0–2400) mL; *p*-value < 0.001) and longer hospital stay (6 (3–25) vs. 5 (2–9) vs. 3 (1–44); *p*-value < 0.001) when compared with the technically minor counterpart. Perioperative outcomes were similar when comparing cirrhotic MILS with the non-cirrhotic cohort.

**Conclusion:**

MILS program implementation can lead to encouraging surgical outcomes even in low- to moderate-volume centers. Although low procedural volume might be predictive of impaired outcomes, long-standing experience in the HPB and liver transplant field could mitigate low-case volume effects on surgical outcomes.

**Supplementary Information:**

The online version contains supplementary material available at 10.1007/s00268-022-06754-z.

## Introduction

The use of MILS has increased dramatically over the past decades [1–3], and, according to the ultimate international guidelines, laparoscopy is now considered the standard of care for minor liver resections [4]. Major laparoscopic liver resections have shown promising outcomes [5–7] recently crowned by the introduction of robotic platforms, which have emerged as viable alternatives [8, 9].

However, the current clinical guidelines are mainly derived from validating perioperative outcomes in experienced high-volume centers. MILS implementation in low- to moderate-volume centers is frequently overlooked resulting in limited data, especially regarding major liver resections [10]. Several factors contribute to maintaining a constant gap between high- and low-volume centers.

The most important contributors are the steepness of the learning curve in liver surgery, which is significantly higher when compared with other minimally invasive procedures, and patient-related elements such as underlying liver cirrhosis [11].

Liver cirrhosis is responsible for adding both intraoperative and postoperative complexity to liver surgery [12]. Most studies highlight how the well-known benefits of MILS might be exploited in this scenario [12–14]. Nevertheless, a minimally invasive approach to cirrhotic liver resection is still a controversial field of study, especially in low-volume centers [15].

The primary aim of this study is to analyze the implementation and outcomes of MILS in one of the largest national Dutch tertiary referral centers, considered a low- to moderate-volume center for liver surgery from a European standpoint. The secondary aim is to analyze the impact of cirrhosis on perioperative outcomes.

## Methods

All consecutive patients who underwent minimally invasive liver surgery at an academic medical center between April 2010 and December 2021 were included. Patient who underwent fenestration of hepatic cysts without any parenchymal transection and resection were excluded. The need for ethical approval was waived by the institutional ethics committee.

All cases were reviewed during weekly multidisciplinary team meeting in which decisions regarding the surgical approach preferred were made according to lesions’ characteristics (e.g., location and size), patients’ performance status and surgeons’ skills. Referring to surgeons’ skills, every MILS performed in our institution is systematically supervised by a senior HPB surgeon with extensive experience in laparoscopic liver surgery. To expand MILS practice and consolidate acquisition of complex laparoscopic skills among younger surgeons, with little experience in HPB surgery, a stepwise learning model is applied. Surgeons at the beginning of the learning curve (first 40 procedure) are supervised on low-difficulty procedure (e.g., technically minor resections) before attempting moderate-difficulty procedure (e.g., minor resections including posteriorly located segments) and so forth. Therefore, major resections will be only attempted by those who successfully overcame the learning curve for both low- and moderate-difficulty procedures.

### Definitions and data collection

Data on all patients were prospectively collected at our Erasmus MC Institutional database, and all analyses were performed retrospectively. Patients were stratified into three study groups according to the difficulty of the resection performed. The resection difficulty was assigned to each case according to the Dutch Liver Collaborative Group (DLCG) definition based on consensus agreements [1, 4, 16].

Major minimally invasive liver resections were defined as any resection of three or more segments. Technically major liver resections were defined as any resection of posteriorly located segments including: segment 7, segment 8, 4a and 1. Every other resection type that did not meet the inclusion criteria for the above-mentioned groups was defined as “technically minor.”

Baseline characteristics for the entire series are listed in Table 1.

**Table 1:** Patients and procedure characteristics

Total Series	Minor		Major	*p-value*
*N* = 212 (100)	*N* = 188 (89)		*N* = 24 (11)	
	Technically Minor	Technically Major		
	N = 177	N = 11		
Age, years, median (IQR)	56 (18–85)	46 (30–73)	51 (21–80)	0.304
Sex, male	67 (38)	5 (46)	7 (29)	0.602
BMI, Kg/m^2^, median (IQR)	26.4 (17.9–45.4)	26.5 (21.0–41.2)	26.2 (18.6–36.4)	0.975
ASA				
I	31 (18)	4 (36)	5 (21)	0.525
II	101 (57)	4 (36)	16 (67)	
III	40 (23)	3 (27)	3 (13)	
IV	4 (2)	–	–	
Diabetes	32 (18)	2 (18)	5 (21)	0.948
Hypertension	50 (28)	4 (36)	6 (25)	0.786
COPD	10 (6)	–	–	0.354
Hepatitis	15 (9)	1 (9)	1 (4)	0.760
Cirrhosis	19 (11)	1 (9)	1 (4)	0.597
Previous abdominal surgery	82 (46)	6 (54)	10 (42)	0.776
Pathology				
Hepatocellular carcinoma	55 (31)	3 (27)	4 (17)	**0.006**
Liver metastases	32 (18)	2 (18)	–	
Benign	80 (45)	4 (36)	18 (75)	
Biliary	10 (6)	2 (18)	2 (8)	
Type of resection				
Non anatomical	34 (19)	–	–	** < 0.001**
Segmentectomy	36 (20)	8 (73)	–	
Left lateral + other segment	1 (1)	3 (27)	–	
Left lateral	106 (60)	–	–	
Left hemihepatectomy	–	–	12 (50)	
Right hemihepatectomy	–	–	12 (50)	
Approach				
Laparoscopic	147 (83)	10 (91)	17 (71)	0.252
Robotic	30 (17)	1 (9)	7 (29)	
Conversion	20 (11)	8 (73)	11 (46)	** < 0.001**
Blood loss, mL, median (IQR)	100 (0–2400)	240 (50–1100)	425 (0–2100)	** < 0.001**
Operative time, minutes, median (IQR)	103 (20–513)	176 (61–347)	297 (60–513)	** < 0.001**
Any morbidity	47 (26)	5 (45)	14 (58)	0.109
Major morbidity	14 (8)	–	3 (13)	0.446
Reoperation rate	4 (2)	–	1 (4)	0.736
Biliary Leakage	5 (3)	–	1 (4)	0.788
Hemorrhage	2 (1)	–	1 (4)	0.458
Liver failure	–	–	1 (4)	**0.02**
Postoperative hospital stay, days, median (IQR)	3 (1–44)	5 (2–9)	6 (3–25)	** < 0.001**
90-day mortality	–	1 (9)	1 (4)	0.083

Bold values represent statistically significant p-values  (< 0.05)

Diabetes was defined as the use of insulin or any antidiabetic drugs preoperatively. Hypertension was defined as the use of any antihypertensive drugs preoperatively. Hepatitis was defined as serologic confirmation of either hepatitis B or C infection. Cirrhosis was defined according to clinical, radiological and laboratory features. Three conversions were conversions to a hand-assisted approach. Considering this small number, the conversion variable was kept binary. All complications within 90 days after surgery were scored and graded according to the Clavien–Dindo classification system [17]. Biliary leakage, hemorrhage and liver failure were scored and graded according to the respective grading systems as proposed by the International Study Group of Liver Surgery [18–20].

Resection margins at final pathology were defined as R0 (tumor free margins) and *R*1 (< 1 mm tumor-free margins).

### Statistical analysis

All categorical variables are shown as numbers with percentages, and differences were tested using chi-square tests. All continuous variables are shown as medians with inter-quartile range (IQR), and differences were tested using Mann–Whitney *U* tests or Kruskal–Wallis test when appropriate.

To identify factors associated with intraoperative conversion, uni- and multivariable logistic regression analyses were performed. A backward selection of all variables with a p-value of 0.100 or lower at univariable analyses was chosen as variable selection method for multivariate analyses. The CUSUM (cumulative sum) method was used to design CUSUM charts for intraoperative blood losses, operative time and conversion. In the CUSUM analyses, the incidence of a chosen event per each case was plotted against the difference between consecutive cases and the expected incidence of the chosen event. The mean values for operative time, blood losses and conversion obtained for the total series were used as expected incidence for the analyses.

All statistical analyses were performed using SPSS Version 26.0 (IBM, Chicago, IL).

## Results

A total of 212 patients were included in the analyses. Cohort characteristics are shown in Table 1. The most common indications for surgery were benign lesions (*n* = 102, 48%) and hepatocellular carcinoma (n = 62, 29%). Liver cirrhosis was present in 21 patients (10%). Major morbidity occurred in 14 patients after technically minor liver resections (7%) and in 3 patients after major liver resections (13%). Reoperation rate was 2% (*n* = 4) after technically minor liver resections and 4% (*n* = 1) after major liver resections. *R*0 resection rate was 99%.

### Difficulty subgroups analyses

Difficulty-based stratification of the entire series resulted in three main comparison groups. Minor liver resections were performed in 188 patients (89%) out of which 177 cases (94%) were defined as “technically minor,” while 11 cases (6%) were defined as “technically major.” The major resection group consisted of 24 cases (11%) with an equal number of left and right hepatectomies. Conversion occurred in 39 procedures (18%) and was more common after technically major resections (n = 8, 73%) when compared to both major and technically minor liver resection (*n* = 11, 46% vs. *n* = 20, 11%; *p* < 0.001). Conversion rates were equal for both robotic and laparoscopic cases (7/38, 18% versus 32/174, 18%; *p* = 1.000) and did not differ for both major and minor liver resection subgroups (data not shown, *p* = 1.000 for both subgroups).

Patients who underwent major liver resections experienced higher intraoperative blood losses (425 (0–2100) vs. 240 (50–1100) vs. 100 (0–2400) mL; *p* < 0.001), longer operative time (297(60–513) vs. 176 (61–347) vs. 103 (20–513) min; *p* < 0.001) and longer length of postoperative hospital stay (6 (3–25) vs. 3 (1–44) vs. 5 (2–9) days; *p* < 0.001) when compared to patients who underwent either technically major or technically minor resections.

No statistically significant differences in major morbidity rates were found. To further investigate the distribution of morbidity among difficulty groups, an additional comparison was performed. Patients were stratified into two distinct difficulty subgroups: major versus minor and technically major vs technically minor liver resections. Overall results did not change considerably from the former analyses. The only noticeable difference was found in overall morbidity rates that were higher in patients undergoing major liver resection when compared to patients undergoing minor liver resection (*n* = 14, 58% vs. *n* = 52, 28%; *p* = 0.005). All results are reported in Supplementary Table 1.

Furthermore, patients undergoing major liver resections reported higher postoperative liver failure rates (*n* = 1, 4% vs. *n* = 0, 0% vs. *n* = 0, 0%; *p* = 0.02) and higher rates of benign liver disease (*n* = 18, 75% vs. *n* = 80, 45% vs. *n* = 4, 36%, *p* = 0.006).

### Risk factors for conversion

Uni- and multivariable logistic regression analyses results are shown in Table 2. At univariate analyses, history of chronic obstructive pulmonary disease (COPB) and higher difficulty of the hepatic resection were identified as risk factors for conversion. At multivariate analyses, history of COPB, higher difficulty of the hepatic resection and preoperative malignant diagnosis were recognized as independent predictors of conversion.

**Table 2:** Uni- and multivariable logistic regression analyses of risk factors contributing to conversion after minimally invasive liver resection

Variable	OR (95% CI)	Univariate analysis for conversion (*p-Value*)	OR (95% CI)	Multivariate analysis for conversion (*p-Value*)
Age, continuous	0.99 (0.98–1.02)	0.941		
Male sex	0.72 (0.36–1.46)	0.367		
BMI, continuous	0.96 (0.89–1.02)	0.243		
ASA*,* (III/IV vs. I/II)	0.64 (0.300–1.38)	0.252		
Diabetes	1.71 (0.75–3.89)	0.200		
Hypertension	0.85 (0.38–1.87)	0.683		
COPD	4.94 (1.36–18.01)	**0.015**	0.11 (0.03–0.44)	**0.002**
Hepatitis	0.57 (0.12–2.60)	0.467		
Cirrhosis	1.05 (0.33–3.31)	0.935		
Previous abdominal surgery	1.45 (0.72–2.92)	0.292		
Malignant diagnosis	1.90 (0.93–3.88)	0.076	0.28 (0.11–0.72)	**0.009**
Difficulty of the resection				
Technically minor	Reference		Reference	
Technically major	14.62 (3.67–58.19)	** < 0.001**	13.90 (4.62–41.82)	** < 0.001**
Anatomically major	4.83 (1.97–11.87)	** < 0.001**	37.01 (8.01–171.04)	** < 0.001**
Approach, (robotic vs. laparoscopic)	1.00 (0.40–2.48)	0.997		

Bold values represent statistically significant p-values  (< 0.05)

### Patients’ characteristics and outcomes in the liver cirrhosis subgroups analyses

The non-cirrhotic cohort consisted of a total of 191 patients (90%). A total of 21 patients (10%) represented the cirrhotic cohort. All cirrhotic patients were classified as Child–Pugh A. Seven had preoperative history of portal hypertension. Clinical characteristics and perioperative outcomes were compared between the two cohorts. Results are shown in Table 3.

**Table 3:** Comparison of patients’ characteristics and perioperative outcomes between patients with and without cirrhosis

	Cirrhosis	No cirrhosis	*p-value*
	*N* = 21	*N* = 191	
Age, years, median (IQR)	66 (61–73)	52 (38–68)	**0.002**
Sex, Male	15 (71)	64 (34)	**0.001**
BMI, Kg/m^2^, median (IQR)	26.9 (23.8–29.5)	26.4 (23.3–30.2)	0.627
ASA			
I	–	40 (21)	** < 0.001**
II	6 (29)	115 (61)	
III	15 (71)	31 (16)	
IV	–	4 (2)	
Diabetes	13 (62)	26 (14)	** < 0.001**
Hypertension	12 (57)	48 (25)	**0.004**
COPD	1 (5)	9 (5)	1.000
Hepatitis	7 (33)	10 (5)	** < 0.001**
Previous abdominal surgery	4 (19)	94 (49)	**0.010**
Pathology			
Hepatocellular carcinoma	16 (76)	45 (24)	**0.001**
Liver metastases	–	35 (18)	
Benign	4 (19)	104 (54)	
Biliary	1 (5)	7 (4)	
Major liver resection	1 (5)	23 (12)	0.480
Type of resection			
Non anatomical	4 (19)	30 (16)	0.742
Segmentectomy	4 (19)	40 (21)	
Left lateral	12 (57)	98 (51)	
Left hemihepatectomy	1 (5)	11 (6)	
Right hemihepatectomy	–	12 (6)	
Difficulty of the resection			
Anatomically major	1 (5)	23 (12)	0.597
Technically major	1 (5)	10 (5)	
Technically minor	19 (90)	158 (83)	
Approach			
Laparoscopic	19 (91)	155 (81)	0.381
Robotic	2 (10)	36 (19)	
Conversion	4 (19)	35 (18)	1.000
Blood loss, mL, median (IQR)	100 (75–400)	100 (20–250)	0.087
Operative time, minutes, median (IQR)	135 (102–199)	108 (80–161)	0.100
Any morbidity	8 (38)	58 (30)	0.279
Major morbidity	2 (10)	15 (8)	0.679
Reoperation rate	–	5 (3)	1.000
Biliary Leakage	–	6 (3)	1.000
Hemorrhage	–	3 (2)	1.000
Liver failure	–	1 (1)	1.000
Postoperative hospital stay, days, median (IQR)	4 (3–7)	3 (3–5)	0.151
90-day mortality	–	2 (1)	1.000

Bold values represent statistically significant p-values  (< 0.05)

Patients within the cirrhotic cohort were more frequently male patients (*n* = 15, 71% vs. *n* = 64, 34%; *p* = 0.002) with higher median age when compared with the non-cirrhotic cohort. Cirrhotic patients reported higher ASA III scores (*n* = 15, 71% vs. *n* = 31, 16%; *p* < 0.001) and higher rates of preoperative comorbidities such as: diabetes (*n* = 13, 62% vs. *n* = 26, 14%; *p* < 0.001), hypertension (*n* = 12, 57% vs. *n* = 48, 25%; *p* = 0.004) and previous history of hepatitis (*n* = 7, 33% vs. *n* = 10, 5%; *p* < 0.001). Perioperative outcomes such as conversion rates, intraoperative blood losses and operative times were similar between the two groups.

### CUSUM analyses

**Fig. 1:**
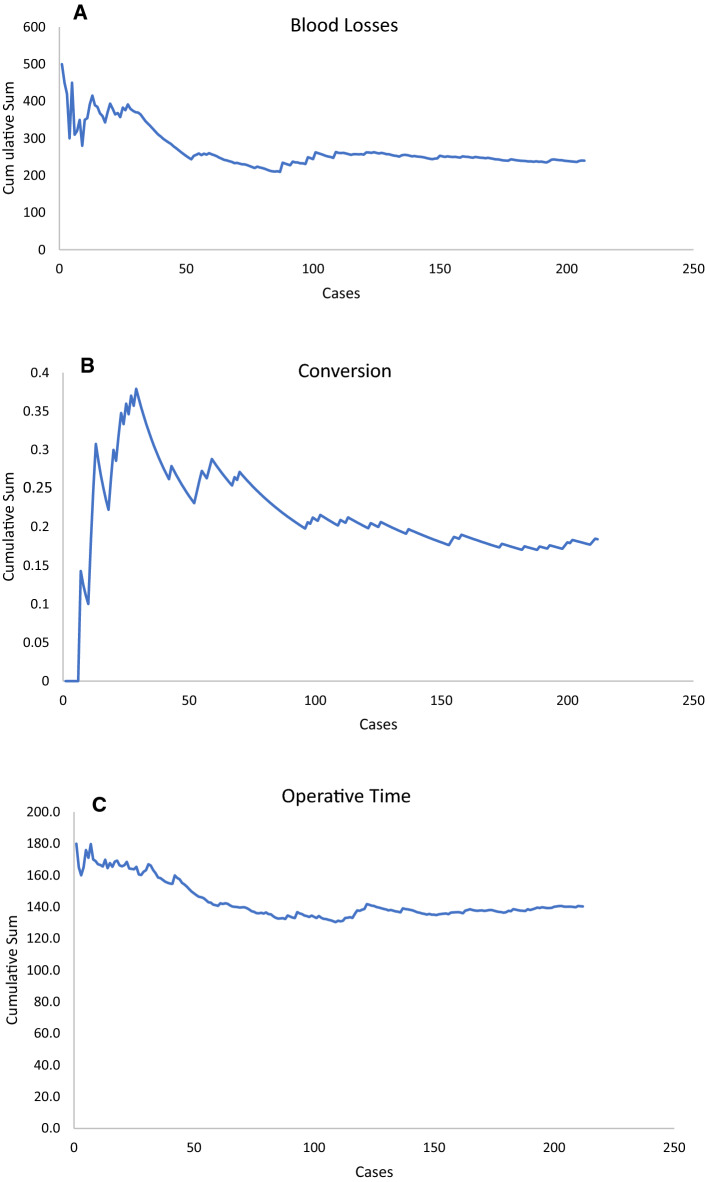
CUSUM learning curves for **a** blood losses, **b** conversion and** c** operative time. The mean values obtained from the entire series, for each one of the three variables analyzed through the CUSUM method, were used as reference values for minimally invasive learning curve plotting

CUSUM analyses’ results are reported in Fig. 1.

Figure 1a shows the learning curve for the entire study series by applying a CUSUM model to intraoperative blood losses. From the 40th hepatic resection onwards, mean intraoperative blood losses progressively decreased and then stabilized after 100 cases. A similar curve was observed when designing CUSUM charts for conversion rates (Fig. 1b).

When performing CUSUM analyses for operative time (Fig. 1c), after the first 40 cases, a continuous downward trend was recorded. This tendency stabilized from the 130th case onwards but remained below the mean operative time for the whole series.

## Discussion

The present study investigated the progressive development of a MILS program in a major Dutch HPB and transplant center. A total of 212 consecutive minimally invasive liver resections over 11 years were analyzed. Patients undergoing major liver resections had higher conversion rates, longer operative time and higher intraoperative blood losses than those within the technically major and minor subgroups. Postoperative morbidity and reoperation rates were comparable between the three groups.

A previous Dutch nationwide study analyzed the implementation of MILS programs and demonstrated that the use of minimally invasive strategies for minor resection is increasing [1]. The above-mentioned paper reported a 10% major morbidity and a 12% conversion rate in 853 minor resections. These results were consistent with the current study.

Nationwide implementation of major liver resection showed slower growth rates, with only 63 procedures performed between 2011 and 2016. Conversion rates for major liver resections were 21%, while major morbidity rates were 14%. Likewise, our series highlighted a gap between the implementation of minor MILS and major MILS with only 24 major liver resections carried out. Conversion rates, within the major group, were 46%, resulting in higher rates when compared to the nationwide data while major morbidity rates were similar (13% vs. 14%). In our series, most conversions occurred during the early years of the MILS program. When no associated contraindications to pneumoperitoneum were present, all early cases were, at least, started in a minimally invasive fashion. Surgeons progressively gained confidence with the different phases of laparoscopic resections even if it was known beforehand that the operation would be completed with a traditional open resection. This upfront strategy helped to systematically develop the program. Its efficacy was further proved in the CUSUM analysis in which conversion rates significantly dropped after roughly 40 procedures.

Still to this day, available literature, regarding the implementation and outcomes of MILS on a national level, is limited. Mainly studies from France [21] and Italy [2, 22] presented insight on this matter. For both studies, results on perioperative outcomes were comparable to our series. Other studies addressed this topic through surveys and questionnaires [23, 24], but few have analyzed the impact of centers’ volume. This subject is erroneously frequently overlooked since extending MILS boundaries into nationwide practice requires MILS implementation in low- to medium-volume centers.

The current study provides detailed data regarding MILS implementation in one of the largest HPB and liver transplant center in the Netherlands, performing on average 20–25 MILS per year. Known as a major Dutch HPB center, it is considered a low- to moderate-volume center from a European standpoint providing a unique perspective. A recent study compared European high-volume centers with low- to medium-volume Dutch centers [10]. Patients undergoing major hepatic resections in the high-volume centers reported conversion rates of 11% and major morbidity rates of 10%, consistent with outcomes observed in our series. However, operative time, intraoperative blood losses and postoperative hospital stay were less in high-volume centers. Nevertheless, the disproportion between the two study groups (*n* = 507 major MILS in high-volume centers vs. *n* = 24 major MILS) makes direct comparison difficult.

This snapshot shows a well-executed MILS implementation for minor resections. Smooth implementation of MILS programs for major resections still remains a challenge.

The learning curve in minimally invasive liver surgery is a popular subject of research [11, 25, 26]. The reported learning curve ranges from 18 to 100 cases, depending on resection type and outcomes. The overall learning curve is 21 cases for minor and 45 cases for major hepatectomy [11]. Moreover, several studies have shown that a higher annual number of minimally invasive liver resections per center is associated with shorter length of stay, lower re-intervention rates and lower conversion rate [10, 27, 28].

Despite positive perioperative outcomes for both anatomically major and technically major liver, the learning curve for major hepatectomy was not matched. This might be explained by the fact that Netherlands population is relatively small compared to those of most European countries leading to lower case volume. In addition, defining the learning curve for MILS as a specific number of resections is debatable [29, 30]. Extensive experience in open liver resections, transplantation and previous training in other complex minimal invasive techniques are important factors that can dramatically influence the steepness of the learning curve and are frequently not considered.

For these reasons, the dichotomy presented by our center in being a national high-volume experienced HPB and transplant center, but a low-volume European center might also be accountable for the satisfactory outcomes observed after major resections which might not be reproducible in nationwide low-volume centers.

This suggested that patients’ outcomes were influenced not only by the annual volume of MILS performed but also by advanced competency in open HPB surgery and liver transplant.

To further prove the above-mentioned concept, we analyzed the impact of liver cirrhosis, a well-known predictor of adverse surgical outcomes, even in expert centers [31], on postoperative outcomes. A total of 21 cirrhotic patients underwent MILS. Their perioperative outcomes were comparable to those of patients without cirrhosis. While liver surgery in cirrhotic patients is very common in Asian center, its numbers are considerably lower in Western countries [32]. Despite the low number of cirrhotic patients in the current study, surgical outcomes were consistent with the ones achieved in international reports [32, 33]. The encouraging results obtained are, again, suggesting that cautious implementation of MILS programs can be promising in low-volume centers.

Finally, the 99% R0 resection rate in patients with malignant lesions was higher when compared with previous series [34, 35]. These promising results demonstrate that the program has always been oncologically safe.

The study has limitations that are mostly related to the retrospective study design. The outcomes should be interpreted in light of the selection of patients for a minimally invasive approach.

In conclusion, this report on 212 minimally invasive liver resection over 11 years of inclusion demonstrates that careful implementation of a MILS program is effective and should be fostered. With careful patient selection, surgical outcomes are acceptable, even in cirrhotic patients. Although procedural volume might be predictive of outcomes, extensive experience in the HPB field and advanced expertise in MILS could attenuate hospital–volume effects on perioperative outcomes.

## Supplementary Information

Below is the link to the electronic supplementary material.

Supplementary file1 (DOCX 13 KB)
