# Supplementary material for: Minimally Invasive Liver Surgery: A Snapshot from a Major Dutch HPB and Transplant Center
Source: World J Surg. 2022 Sep 26;46(12):3090–9. doi: 10.1007/s00268-022-06754-z (PMC9636118; doi:10.1007/s00268-022-06754-z)
Supplement: Supplementary file 1 — Supplementary file1 (DOCX 13 KB) [file 268_2022_6754_MOESM1_ESM.docx]

| **Total Series**  N = 212 (100) | **Minor**  N = 188 | **Major**  N = 24 | *P-value* | **Technically Minor** | **Technically Major** | *P-value* |
| --- | --- | --- | --- | --- | --- | --- |
| **Any morbidity** | 52 (28) | 14 (58) | **0.005** | 47 (26) | 5 (45) | 0.180 |
| **Major morbidity** | 14 (7) | 3 (13) | 0.417 | 14 (8) | 0 (0) | 1.000 |

**Supplementary Table 1**: Additional comparison between difficulty subgroups for morbidity
